# Activities of daily living limitations and family doctor contract services among overweight and obese older adults: is there a rural-urban difference?

**DOI:** 10.1186/s12875-023-02177-4

**Published:** 2023-10-28

**Authors:** Jingjing Luo, Dan Zhao, Tingting Gao, Jingjie Sun, Peilong Li, Xuehong Wang, Xueqing Wang, Shujun Chai, Jiayan Li, Chengchao Zhou

**Affiliations:** 1 Centre for Health Management and Policy Research, School of Public Health, Cheeloo College of Medicine, Shandong University, 44 Wen-hua-xi Road, 250012 Jinan, China; 2 Institute of Health and Elderly Care, Shandong University, 250012 Jinan, China; 3 Shandong Health Commission Medical Management Service Center, 250012 Jinan, China; 4 NHC Key Lab of Health Economics and Policy Research, Shandong University), 250012 Jinan, China

**Keywords:** ADL limitations, Family doctor contract services, Overweight/obesity

## Abstract

**Background:**

The purpose of this study was to evaluate the relationship between activities of daily living (ADL) limitations and family doctor contract services among overweight and obese older adults, as well as to examine whether this association varies by urban-rural residence.

**Methods:**

Data for the present study were obtained from the sixth Health Service of Shandong province, China. A total of 4,249 overweight and obese older adults were included in this study. Binary logistic regression models were used to evaluate the relationship between ADL limitations and family doctor contract services, to examine the potential differences between urban and rural areas.

**Results:**

Of 4,249 overweight and obese older adults, the prevalence of limitations in ADL and family doctor service contracting rate in Shandong province were 12.47% and 66.46%, respectively. The results of the regression analyses revealed that overweight and obese older adults with ADL limitations were more likely to sign up for family doctor services than those without ADL limitations (OR = 1.27, 95%CI: 1.02–1.58, *P* = 0.033). Only among rural overweight and obese older adults, the relationship between ADL limitations and family doctor contract services was statistically significant (OR = 1.50, 95%CI: 1.13–1.99, *P* = 0.005).

**Conclusions:**

Our study found a significant association between ADL limitations and family doctor contract services among overweight and obese older adults in Shandong, China. This relationship differed by urban-rural residence. To promote the positive development of the family doctor contract service system, the government should increase publicity, provide personalized contracted services, and prioritize the healthcare needs of overweight and obese older adults with ADL limitations, with special attention to rural areas.

**Supplementary Information:**

The online version contains supplementary material available at 10.1186/s12875-023-02177-4.

## Introduction

In the context of global population aging, the trend of deep aging of China’s population is emerging [1]. Based on the provisions of Law of the People’s Republic of China on the Protection of the Rights and Interests of the Elderly, this study defined older adults as people who were 60 years old and above [2]. China’s population aged 60 years and above is expected to account for 20% of the total population by 2035 [3]. Healthy aging is the least costly and most effective way for China to deal with population aging [4]. For the older population, healthy aging is more important than merely surviving old age. Even though most countries have taken actions to promote healthy aging [5], there are still insufficient supplies of institutions, workforce, services, and policies compared to the health needs of older adults, and many serious health problems are still prevalent among the older population.

Overweight and obesity have become a public health issue that cannot be ignored in older adults [6]. The Fifth Bulletin of National Physique Monitoring revealed that in 2020, overweight and obesity rates among older adults in China were 41.7% and 16.7%, respectively [7]. Overweight and obese older adults have a higher risk of chronic non-communicable diseases such as cardiovascular and cerebrovascular diseases, diabetes, and hypertension due to the decline in basic metabolic function and excessive fat accumulation in the body [6, 8]. Furthermore, previous studies have shown that overweight and obesity may increase the risk of psychological problems, decreased physical functioning, and mortality in older adults [9, 10]. This may lead to an increased economic burden of disease and reduced quality of life in older adults, posing a huge potential challenge to the healthcare system in China [6]. As a result, more health care and health management services for overweight and obese older adults are required, which is especially important to prevent chronic diseases and promote healthy aging.

The family doctor system, which originated in the United Kingdom, has been promoted globally since the 1970 and 1980 s. Family doctor plays the role of a health “gatekeeper” [11]. As of 2015, more than 50 countries and regions around the world had implemented the family doctor service system [12]. As a primary care service model, family doctor contract services have obvious advantages in terms of health management, disease prevention and control, rationalizing health resources, and lowering medical costs [13, 14]. As a developing country, China has been establishing a system of contracted family doctor services since 2009 [15]. In May 2016, the State Council issued the Guiding Opinions on Promoting Family Doctor Contract Services, marking the official implementation of family doctor contract services in China [16]. Shandong province issued the Implementation Advice on Accelerating and Improving Family Doctor Contract Services in December 2016, which began to comprehensively promote the family doctor contract service system [17]. Older adults, the disabled, and patients with chronic diseases such as hypertension and diabetes were classified as the priority population. By 2020, we will strive to expand the contracted services to the whole population and basically achieve the full coverage of the family doctor contract service system [17]. Residents and family doctors signed voluntarily in Shandong province [18]. Family doctors provide services such as diagnosis and treatment of common diseases, preventative health care, and health management to the contracted residents, which improves their health [17, 18]. China’s family doctor contracting system is still at its early stage, with issues such as uneven service quality and a low personalized contracting rate, especially the lack of precise contract services for key populations such as overweight and obese older people [16, 17, 19]. It is necessary to further explore the influencing factors of family doctor contract services among overweight and obese older adults, to provide an empirical foundation for optimizing the family doctor contract service system and effectively satisfy older adults’ health needs.

The ability to perform activities of daily living (ADL) in the older population gradually declines as they age [20]. ADL limitations raise the cost and financial burden of medical care for older people [21], leading to a decline in their quality of life [22]. The prevalence of limitations in ADL is higher in overweight and obese older adults [10, 20, 23]. Overweight and obese older adults with ADL limitations were at higher risk for chronic diseases and require more access to health care and disease management services [21, 24, 25]. Due to limited mobility, overweight and obese older adults with ADL limitations do not have timely access to health services, resulting in worsening or even deteriorating of their conditions [20, 26]. Family doctor teams can provide proactive, continuous, and comprehensive health management services to overweight and obese older adults with ADL limitations, as well as develop personalized life interventions to effectively prevent and control diseases [27]. Previous studies have shown an association between ADL limitations and family doctor contract service [28–30]. Older adults with ADL limitations may be more disposed to receive regular home visits from their family physicians [28, 31]and have higher contracting rates for family doctor services [28, 29]. However, it is unclear whether there is a correlation between ADL limitations and family doctor contract services among overweight and obese older adults.

Additionally, rural and urban areas in China represent two different social statuses [32]. Compared to urban residents, rural residents are more likely to be farmers with lower levels of education and income [33], less community infrastructure, and relatively less access to government-sponsored public resources or healthcare services [34]. Previous studies indicated that rural older adults had a higher rate of signing up for family doctor services than urban older adults [35, 36]. Simultaneously, the resource-limited environment increases the risk of health problems in older adults [37]. Prior research found that rural older adults had a higher risk of ADL limitations than their urban counterparts [38, 39]. The above urban-rural differences may affect the relationship between ADL limitations and family doctor contract services. To date, there are no studies to examine the rural-urban disparity in the relationship between ADL limitations and family doctor contract services.

Based on the above background, the present study aims to investigate the relationship between ADL limitations and family doctor contract services among overweight and obese older adults, as well as whether there is a rural-urban difference in this correlation. Overall, we proposed the following specific hypotheses: (1) Hypothesis 1: ADL limitations are associated with higher contracting rates for family doctor services among overweight and obese older adults in Shandong, China. (2) Hypothesis 2: The relationship between ADL limitations and family doctor contract services varies in urban and rural areas. The findings of this study could contribute to optimizing the family doctor contract service system, alleviate healthcare system stress, and promote healthy aging.

## Methods

### Data source and participants

The data for this study were derived from the sixth Health Service Survey of Shandong Province in 2018. A multi-stage stratified cluster random sampling method was employed to cover 17 cities in the province under the leadership of the Statistical Information Center of the National Health Commission and the Health Commission of Shandong province [40]. First, twenty counties (districts) were randomly selected from a total of 137 counties (districts) as study sites in Shandong province. Second, five townships were randomly selected from each sampled county. Third, we randomly selected 2 villages (neighborhood committees) from each selected township. A total of 20 counties (districts), 100 townships, and 200 villages (communities), with a total of 12,938 households and 35,264 respondents, were finally sampled to complete this survey.

Data were collected face-to-face by well-trained investigators using a structured questionnaire with the informed consent of respondents. Given our focus on a sample of overweight and obese older adults, participants who met the following exclusion criteria were excluded from this study: (1) participants aged < 60 years (n = 26,361), (2) respondents with serious mental illnesses such as dementia (n = 261), and (3) respondents who were not overweight and obese [Body mass index (BMI) < 24.0 kg/m2 (n = 4,353)] [41]. BMI, which is calculated as weight in kilograms divided by the square of height in meters, is used to determine overweight and obesity. Weight and height were measured using the “anthropometric method” by trained technicians [42]. Participants were measured by a stadiometer and weighing scale while wearing light clothing and no shoes [43]. BMI was categorized into four categories: underweight (BMI<18.5), normal (18.5 ≤ BMI<24.0), overweight (24.0 ≤ BMI<28.0), and obesity (BMI ≥ 28.0) [41]. In addition, to obtain complete and accurate data, we excluded participants with missing values for ADL limitations and household income (n = 40). Finally, the study sample consisted of 4,249 overweight and obese older adults. Figure S1 appears in a flowchart of the sample for this study.

### Measures

#### Family doctor contract services

Participants were asked “Have you contracted for family doctor services?” and could answer with “yes” or “no”. Family doctor contract services were categorized as a dichotomous variable: the contract code was 1, and the uncontracted code was 0.

#### Activities of daily living

To examine activities of daily living (ADL), we used the Katz Index Scale, which included six items: dressing, eating, bathing or showering, getting in or out of bed, toileting, and controlling urination and defecation [44]. Answers to each item were given on a 4-point scale (1 = no difficulty; 2 = difficulty, but can still do it independently; 3 = difficulty and needs help; 4 = Unable to complete). If the answer to any of the items was “difficulty and need help” or “unable to complete”, the older person was classified as having limitations in ADL. ADL limitations were eventually coded as a dichotomous variable (yes, no). The option of “yes” indicated an ADL limitation, while “no” implied no ADL dysfunction. The Chinese version was used to assess the functional limitations of subjects, which has been shown to have good reliability and validity [45].

#### Covariates

We considered several potential confounding variables in our analysis, including demographic characteristics, lifestyle behaviors, health status, and healthcare accessibility. Sociodemographic characteristics included gender (male or female), age (years), residence (urban or rural area), the highest education level (illiterate, primary school, middle school, and high school or above), marital status [single (unmarried, divorced, widowed), married], and household income (four types based on percentile, with the 1st quartile being the worst and the 4th quartile being the richest). Household income was measured by asking the total income of the respondent’s household in the previous year (2017). Health security included medical insurance (yes or no) and accessibility to health services. Accessibility to health services, measured by a question: “How many minutes does it take to get from your home to the nearest health care facility (the most accessible mode of transportation)?” Based on this question, we divided the time into ≤ 10 min or > 10 min. Lifestyle behavioral factors included smoking (yes or no) and drinking alcohol (yes or no). Health status was assessed by asking participants about their self-rated health and chronic health status. Self-rated health was divided into five categories (very good, good, fair, poor, and very poor). Self-reported chronic diseases fall into three categories (no chronic disease, one chronic disease, and multimorbidity).

### Statistical analysis

For all statistical analyses, Stata 15.1 (Stata Corp, College Station, TX, USA) was used. First, we described the characteristics of continuous and categorical variables using mean (SD) or frequency (percentage), respectively. Subsequently, chi-square tests (for categorical variables) and Student’s t-tests (for continuous variables) were used to compare the individual characteristics of overweight and obese older adults who had signed up for family doctor contract services versus those who had not contracted. Second, binary logistic regression was employed to investigate the relationship between ADL limitations and family doctor contract services. Several progressive models were evaluated. Model 1 was unadjusted. Model 2 adjusted for all confounding factors, including demographic characteristics, lifestyle behaviors, health security, and health status. Third, Model 3 was based on Model 2, incorporating the interaction term ADL limitations × residence, to test the interaction effect of ADL limitations and urban-rural residence on family doctor contract services further. Meanwhile, we performed a stratified analysis of the residence to explore possible differences between subgroups. The reported odds ratio (OR) with 95% confidence intervals (CIs) was calculated, and statistical significance was set as a two-tailed *p*-value < 0.05.

## Results

### Characteristics of the participants

Table 1 shows the demographic characteristics of the participants. The final sample consisted of 4,249 overweight and obese participants with a mean age of 67.73 years (SD = 6.09), of whom 46.39% were male, 48.93% were rural residents, 86.63% were married, and 73.76% had primary education or above. Of all respondents, 66.46% reported contracting for family doctor services. In general, compared to overweight and obese seniors who did not have contracted family doctor services, the majority of those who signed up were older, were female, had lower household income, and had more multiple diseases and ADL limitations. In this study, the rate of ADL limitations among overweight and obese older adults was 12.47%.

**Table 1:** Basic characteristic of the participants among overweight and obese older adults

Characteristic	N (%)	Family doctor contract services		*P*-value
		Yes (%)	No (%)	
Observations	4249	2824 (66.46)	1425 (33.54)	
Age (years), mean (*SD*)	67.73 (6.09)	67.94 (5.98)	67.32 (6.30)	**0.002**
Gender				0.398
Male	1971 (46.39)	1297 (45.93)	674 (47.30)	
Female	2278 (53.61)	1527 (54.07)	751 (52.70)	
Residence				**< 0.001**
Urban	2170 (51.07)	1534 (54.32)	636 (44.63)	
Rural	2079 (48.93)	1290 (45.68)	789 (55.37)	
Education				**< 0.001**
Illiterate	1115 (26.24)	805 (28.51)	310 (21.75)	
Primary school	1381 (32.50)	911 (32.26)	470 (32.98)	
Middle school	1102 (25.94)	688 (24.36)	414 (29.05)	
High school and above	651 (15.32)	420 (14.87)	231 (16.21)	
Marital status				0.166
Single ^a^	568 (13.37)	363 (12.85)	205 (14.39)	
Married	3681 (86.63)	2461 (87.15)	1220 (85.61)	
Household income ^b^				**0.001**
Q1	1319 (31.04)	872 (30.88)	447 (31.37)	
Q2	806 (18.97)	570 (20.18)	236 (16.56)	
Q3	1095 (25.77)	742 (26.27)	353 (24.77)	
Q4	1029 (24.22)	640 (22.66)	389 (27.30)	
Medical insurance				**< 0.001**
Yes	4204 (98.94)	2807 (99.40)	1397 (98.04)	
No	45 (1.06)	17 (0.60)	28 (1.96)	
The time to the nearest clinic (minutes)				0.492
≤ 10	3727 (87.71)	2484 (87.96)	1243 (87.23)	
>10	522 (12.29)	340 (12.04)	182 (12.77)	
Smoking				**< 0.001**
Yes	642 (15.11)	384 (13.60)	258 (18.11)	
No	3607 (84.89)	2440 (86.40)	1167 (81.89)	
Drinking				0.892
Yes	1205 (28.36)	799 (28.29)	406 (28.49)	
No	3044 (71.64)	2025 (71.71)	1019 (71.51)	
Self-rated health				0.323
Very good	1251 (29.44)	809 (28.65)	442 (31.02)	
Good	723 (17.02)	501 (17.74)	222 (15.58)	
Fair	1138 (26.78)	754 (26.70)	384 (26.95)	
Poor	667 (15.70)	449 (15.90)	218 (15.30)	
Very poor	470 (11.06)	311 (11.01)	159 (11.16)	
Chronic health conditions				**< 0.001**
No chronic condition	1622 (38.17)	1003 (35.52)	619 (43.44)	
One chronic condition	1561 (36.74)	1067 (37.78)	494 (34.67)	
Multimorbidity	1066 (25.09)	754 (26.70)	312 (21.89)	
Activities of daily living limitations				**0.006**
Yes	530 (12.47)	380 (13.46)	150 (10.53)	
No	3719 (87.53)	2444 (86.54)	1275 (89.47)	

Note:

^a^ Singles include those who are unmarried (35, 0.82%), divorced (14, 0.33%) and widowed (519, 12.21%);

^b^ Quartile 1 (Q1) was the poorest and Quartile 4 (Q4) was the richest

The total percentage may not equal to 100 due to rounding;

Abbreviations: SD = Standard deviation

### Association between ADL limitations and family doctor contract services

The results of the binary logistic regression analysis are shown in Table 2. In Model 1, there was a significant positive correlation between ADL limitations and contracted family doctor services among overweight and obese older adults (OR = 1.32, 95%CI: 1.08–1.62, *P* = 0.006). The rate of signing up for family doctor services for overweight and obese older adults with ADL limitations was 1.32 times higher than those without ADL limitations. After accounting for the selection and control of recognized factors influencing the contracting of family doctor services, the results of Model 2 showed that a significantly higher proportion of participants with ADL limitations contracted for family doctor services, which was 1.27 times higher than that of those without ADL limitations (OR = 1.27, 95%CI: 1.02–1.58, *P* = 0.033).

**Table 2:** Association between ADL limitations and family doctor contract services

Characteristics	Model 1^a^		Model 2^b^		Model 3^c^	
	OR (95% CI)	*P*-value	OR (95% CI)	*P*-value	OR (95% CI)	*P*-value
*Main terms*						
ADL limitations (No ^Ref^)						
Yes	1.32 (1.08, 1.62)	**0.006**	1.27 (1.02, 1.58)	**0.033**	0.57 (0.29, 1.15)	0.118
Residence (Urban ^Ref^)						
Rural			0.59 (0.51, 0.68)	**< 0.001**	0.56 (0.48, 0.65)	**< 0.001**
*Interaction term*						
ADL limitations × Residence (Without ADL limitations × Urban ^Ref^)						
ADL limitations × Rural					1.64 (1.08, 2.48)	**0.020**
*Controls*						
Age			1.01 (1.00, 1.03)	**0.023**	1.01 (1.00, 1.03)	**0.018**
Gender (Male ^Ref^)						
Female			0.90 (0.76, 1.08)	0.254	0.90 (0.77, 1.07)	0.246
Education (Illiterate ^Ref^)						
Primary school			0.72 (0.60, 0.86)	**< 0.001**	0.72 (0.60, 0.87)	**< 0.001**
Middle school			0.62 (0.50, 0.76)	**< 0.001**	0.62 (0.50, 0.76)	**< 0.001**
High school and above			0.64 (0.50, 0.82)	**< 0.001**	0.64 (0.50, 0.82)	**< 0.001**
Marital status ^d^ (Single ^Ref^)						
Married			1.33 (1.09, 1.62)	**0.006**	1.33 (1.09, 1.63)	**0.005**
Household income ^e^ (Q1 ^Ref^)						
Q2			1.21 (0.99, 1.47)	0.063	1.20 (0.99, 1.47)	0.066
Q3			1.01 (0.84, 1.22)	0.902	1.01 (0.84, 1.21)	0.931
Q4			0.70 (0.57, 0.86)	**0.001**	0.70 (0.57, 0.85)	**< 0.001**
Medical insurance (No ^Ref^)						
Yes			3.57 (1.93, 6.62)	**< 0.001**	3.61 (1.94, 6.69)	**< 0.001**
The time to the nearest clinic (minutes) (≤ 10 ^Ref^)						
>10			0.88 (0.72, 1.07)	0.203	0.88 (0.72, 1.07)	0.200
Smoking (No ^Ref^)						
Yes			0.72 (0.59, 0.89)	**0.002**	0.73 (0.60, 0.89)	**0.002**
Drinking (No ^Ref^)						
Yes			1.13 (0.95, 1.35)	0.173	1.13 (0.94, 1.35)	0.186
Self-rated health (Very good ^Ref^)						
Good			1.40 (1.14, 1.72)	**0.001**	1.40 (1.14, 1.73)	**0.001**
Fair			1.29 (1.07, 1.55)	**0.008**	1.29 (1.08, 1.56)	**0.006**
Poor			1.46 (1.16, 1.81)	**0.001**	1.45 (1.16, 1.80)	**0.001**
Very poor			1.43 (1.12, 1.82)	**0.004**	1.43 (1.12, 1.82)	**0.004**
Chronic health conditions (No chronic condition ^Ref^)						
One chronic condition			1.38 (1.18, 1.60)	**< 0.001**	1.37 (1.18, 1.60)	**< 0.001**
Multimorbidity			1.57 (1.31, 1.88)	**< 0.001**	1.57 (1.31, 1.88)	**< 0.001**

Note:

Statistically significant *p* < 0.05 values are indicated in bold

^a^ Model 1: Unadjusted;

^b^ Model 2: Additionally adjusted for gender, residence, age, education, marriage status, household income, medical insurance, the time to the nearest clinic, smoking, drinking, self-rated health and chronic health conditions;

^c^ Model 3: Adjusted for model 2 criteria and the interaction between activities of daily living and residence;

^d^ Singles include those who are unmarried (35, 0.82%), divorced (14, 0.33%) and widowed (519, 12.21%);

^e^ Quartile 1 (Q1) was the poorest and Quartile 4 (Q4) was the richest

Abbreviations: ADL = Activities of daily living; CI = Confidence interval; OR = odds ratio

### Effect of residence on the association between ADL limitations and family doctor contract services

Model 3 illustrates the interaction effects of ADL limitations and residence on family doctor contract services after controlling for a broad set of covariates. In a fully adjusted model, there was significant interaction in ADL limitations × residence with family doctor contract services (OR = 1.64, 95%CI: 1.08–2.48, *P* = 0.020). As shown in Table 3, the relationship between ADL limitations and family doctor contract services was stratified by urban-rural residence. ADL limitations were only associated with higher contracting rates for family physician services among overweight and obese rural older adults (OR = 1.50, 95%CI: 1.13–1.99, *P* = 0.005).

**Table 3:** Association between ADL limitations and family doctor contract services stratified by residence

Variable	Urban (n = 2170)				Rural (n = 2079)			
	Model 1^a^		Model 2^b^		Model 1^a^		Model 2^b^	
	OR (95% CI)	*P*-value	OR (95% CI)	*P*-value	OR (95% CI)	*P*-value	OR (95% CI)	*P*-value
ADL limitations								
No	1.00 (reference)		1.00 (reference)		1.00 (reference)		1.00 (reference)	
Yes	1.06 (0.78, 1.45)	0.716	0.89 (0.63, 1.25)	0.494	1.66 (1.28, 2.16)	**< 0.001**	1.50 (1.13, 1.99)	**0.005**

Note:

^a^ Model 1 was unadjusted;

^b^ Model 2 was adjusted for gender, age, education, marriage status, household income, medical insurance, the time to the nearest clinic, smoking, drinking, self-rated health and chronic health conditions;

Abbreviations: ADL = Activities of daily living;

Statistically significant *p* < 0.05 values are indicated in bold

## Discussion

To the best of our knowledge, this is the first study to explore urban-rural disparities in the relationship between ADL limitations and family doctor contract services among overweight and obese older adults. This study will provide a new perspective on improving family doctor contract service policies and optimizing the rational allocation of health resources. Our findings suggested that ADL limitations were associated with higher rates of contracting for family doctor services among overweight and obese older adults, as well as urban-rural differences in this relationship. Specifically, a significant positive association between ADL limitations and family doctor contract services relationship was observed only in overweight and obese older adults living in rural areas, but not in those living in urban areas.

This study revealed that ADL limitations were positively associated with signing up for family doctor services among overweight and obese older adults in Shandong, China. Previous studies found that older adults with ADL limitations sign up for family doctor services at a significantly higher rate than their peers, which is consistent with our findings [28–30, 46]. Several possible explanations for this finding are as follows. First, overweight and obese older adults with ADL limitations may face several barriers to receiving needed healthcare services, such as transportation and body image pressures [47, 48]. Family doctors can carry out free home service programs for contracted people with limited mobility to meet the medical service needs of older adults with ADL limitations [27]. Second, this may be related to differences in social participation. Numerous studies have shown that overweight and obesity, as well as ADL limitations, could reduce social participation in older adults [49, 50], resulting in social integration difficulties. Reduced social participation can be detrimental to older adults’ physical and mental health, as well as their well-being in life [51]. On the one hand, family physicians can improve residents’ proper understanding and acceptance of overweight and obese older adults with ADL limitations through community health education [16, 27]. Family doctors, on the other hand, can improve the self-efficacy and social participation of overweight and obese older adults with ADL limitations through programs like rehabilitation guidance and social adaptation enhancement to better integrate them into the community [13, 16, 27].

We found that the relationship between ADL limitations and family doctor contract services differed between urban and rural subgroups among overweight and obese older adults, with only a negative association among rural older adults. One possible reason was that being overweight and obese, as well as ADL limitations, were found to have a more substantial detrimental influence on the physical and mental health of older adults [9, 21, 24, 25]. Family physicians can provide specialized health care services to overweight and obese older adults with ADL limitations to prevent disease and improve their health and quality of life [16, 27]. China has a dual economic structure, with highly developed industrial sectors in cities and relatively backward agricultural sectors in rural areas co-existing [32, 52]. This creates a division in social economy and family structures between rural and urban China [52]. On the one hand, due to the difference in economic income levels, urban residents with a higher economic base may be more inclined to seek better and finely specialized medical services rather than being limited to contracted services provided by family doctors [53]. In comparison to overweight and obese urban older adults with ADL limitations who have higher requirements for the technical level of doctors, these rural older adults may subjectively recognize the technical level and service capability of the family doctor contract service team and are more willing to sign up. On the other hand, nuclear families are becoming more common in urban areas, while the extended family remains the preferred family model in rural China [54]. Rural overweight and obese older adults with ADL limitations are more likely to receive informal care from family members or other relatives. However, informal caregivers frequently lack specialized caregiving knowledge and skills, which can harm the health of the care recipients [55, 56]. Thus, rural overweight and obese older adults with ADL limitations have a greater need for family physicians to provide them with specialized care services as well as skill training for their family caregivers, thereby improving their quality of life and well-being in later life [27].

The findings of this study provided a novel intervention perspective for optimizing the family doctor contract service system and promoting healthy aging. First, the government should continue to increase policy support, improve public service facility accessibility, and implement targeted protection policies for older adults with ADL limitations. Meanwhile, it was necessary to increase subsidies for ADL dysfunction monitoring and medication. Furthermore, the government should provide more personalized family doctor contract service programs to meet the actual needs of overweight and obese older adults with ADL limitations and improve their quality of life. Finally, primary health care should strengthen screening for ADL limitations, as well as increase targeted publicity and health education for overweight and obese older adults. However, there are some potential challenges to implementing these policies. First, despite the Chinese government has efforts to promote the family doctor service policy, residents’ knowledge of the policy remains limited [57]. Second, primary health care institutions lack high-quality medical resources and qualified family doctors [58]. These are the most important factors affecting the willingness of older adults to sign service contracts [58, 59].

Several limitations of this study should be considered when interpreting the findings. First, this was a cross-sectional study, and the association between ADL limitations and family doctor contract services could not be interpreted as a causal effect. In future studies, the above-mentioned relationships can be investigated using a longitudinal design. Second, the variables in this study were self-reported, which may have contributed to recall bias. Finally, this study was only conducted in Shandong Province, and the generalizability of the main findings must be confirmed in China.

## Conclusions

The current study found that ADL limitations were associated with higher rates of contracting for family doctor services among overweight and obese older adults, as well as differences in this association between rural and urban areas. Only in rural overweight and obese older adults, there was a statistically significant relationship between ADL limitations and family doctor contract services. The government should prioritize meeting the healthcare needs of overweight and obese older adults living in rural areas with ADL limitations, promoting the sustainable development of the family doctor contract service system, and improving the well-being of older adults in China.

### Electronic supplementary material

Below is the link to the electronic supplementary material.

Supplementary Material 1

## Data Availability

The datasets used in the current study are not publicly available due to the confidential policy but are available from the corresponding author on reasonable request.

## Abbreviations

ADL: Activities of daily living
BMI: Body mass index
