# Supplementary figures and images for: Activities of daily living limitations and family doctor contract services among overweight and obese older adults: is there a rural-urban difference?
Source: BMC Prim Care. 2023 Oct 28;24:223. doi: 10.1186/s12875-023-02177-4 (PMC10612158; doi:10.1186/s12875-023-02177-4)

**Supplementary material**


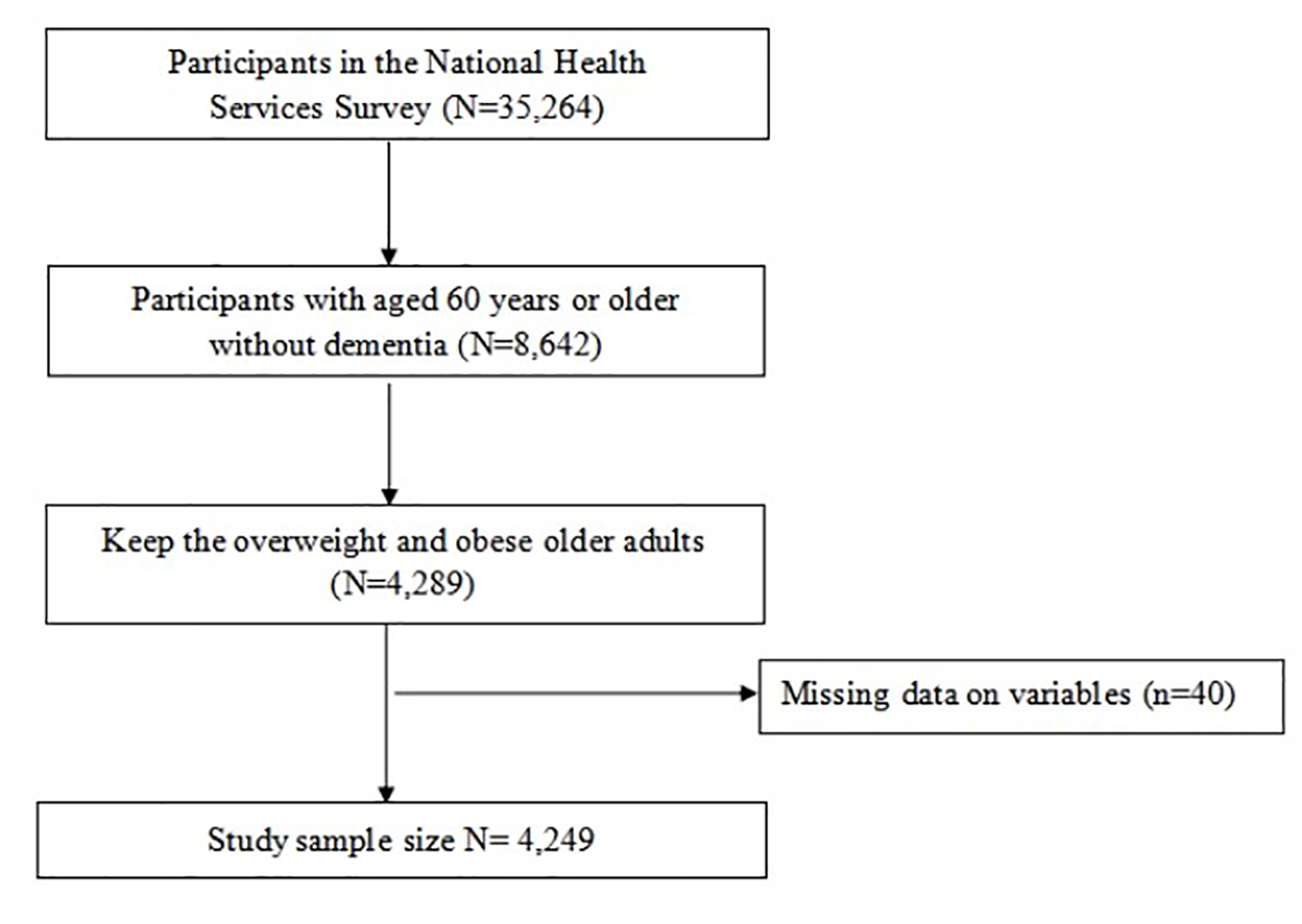


**Figure S1 Flow of participants into study sample.**

Supplement: Supplementary file 1 — Supplementary Material 1 [file 12875_2023_2177_MOESM1_ESM.doc]
